# CFD Modeling of Chamber Filling in a Micro-Biosensor for Protein Detection

**DOI:** 10.3390/bios7040045

**Published:** 2017-10-03

**Authors:** Meiirbek Islamov, Marzhan Sypabekova, Damira Kanayeva, Luis Rojas-Solórzano

**Affiliations:** 1 Department of Chemical Engineering, Nazarbayev University, Astana 010000, Kazakhstan; meiirbek.islamov@nu.edu.kz; 2 Graduate Program in Science, Engineering, and Technology & National Laboratory Astana, Nazarbayev University, Astana 010000, Kazakhstan; msypabekova@nu.edu.kz; 3 Department of Biology, School of Science and Technology, Nazarbayev University, Astana 010000, Kazakhstan; dkanayeva@nu.edu.kz; 4 Department of Mechanical Engineering, Nazarbayev University, Astana 010000, Kazakhstan

**Keywords:** tuberculosis, biosensor, microfluidic cell, multiphase flow, Computational Fluid Dynamics (CFD), ANSYS-CFX

## Abstract

Tuberculosis (TB) remains one of the main causes of human death around the globe. The mortality rate for patients infected with active TB goes beyond 50% when not diagnosed. Rapid and accurate diagnostics coupled with further prompt treatment of the disease is the cornerstone for controlling TB outbreaks. To reduce this burden, the existing gap between detection and treatment must be addressed, and dedicated diagnostic tools such as biosensors should be developed. A biosensor is a sensing micro-device that consists of a biological sensing element and a transducer part to produce signals in proportion to quantitative information about the binding event. The micro-biosensor cell considered in this investigation is designed to operate based on aptamers as recognition elements against *Mycobacterium tuberculosis* secreted protein MPT64, combined in a microfluidic-chamber with inlet and outlet connections. The microfluidic cell is a miniaturized platform with valuable advantages such as low cost of analysis with low reagent consumption, reduced sample volume, and shortened processing time with enhanced analytical capability. The main purpose of this study is to assess the flooding characteristics of the encapsulated microfluidic cell of an existing micro-biosensor using Computational Fluid Dynamics (CFD) techniques. The main challenge in the design of the microfluidic cell lies in the extraction of entrained air bubbles, which may remain after the filling process is completed, dramatically affecting the performance of the sensing element. In this work, a CFD model was developed on the platform ANSYS-CFX using the finite volume method to discretize the domain and solving the Navier–Stokes equations for both air and water in a Eulerian framework. Second-order space discretization scheme and second-order Euler Backward time discretization were used in the numerical treatment of the equations. For a given inlet–outlet diameter and dimensions of an in-house built cell chamber, different inlet liquid flow rates were explored to determine an appropriate flow condition to guarantee an effective venting of the air while filling the chamber. The numerical model depicted free surface waves as promoters of air entrainment that ultimately may explain the significant amount of air content in the chamber observed in preliminary tests after the filling process is completed. Results demonstrated that for the present design, against the intuition, the chamber must be filled with liquid at a modest flow rate to minimize free surface waviness during the flooding stage of the chamber.

## 1. Introduction

**Figure 1:**
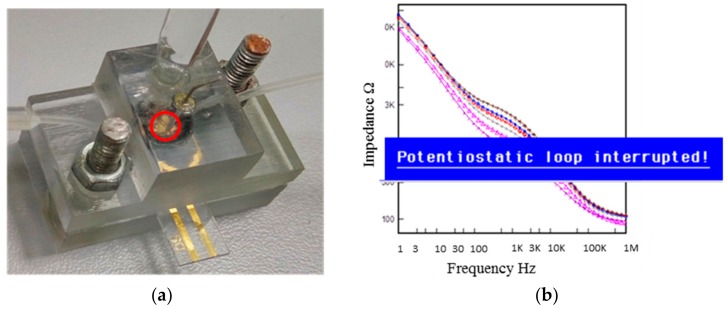
(**a**) Assembled microfluidic device with reference, counter and working electrodes. The bottom and top parts of the device were mounted via bolts and the working area was fixed using an o-ring. The inlet and outlet tubes were positioned at 45° from the sensing surface. The inlet tube was connected to the pumping system, which permitted the liquid flow at a specified flow rate. The red circle encloses the area where air bubbles formed during the course of the experiment. (**b**) Error message obtained during impedance measurement: “Potentiostatic loop interrupted.” In the background, typical data for impedance vs. frequency measurement after protein binding.

*Mycobacterium tuberculosis* (*Mtb*) is a dangerous pathogenic bacterium that causes Tuberculosis (TB), which is one of the leading causes of death from infectious diseases in the world. The rapid and early diagnosis, as well as its treatment, is crucial for the effective control of TB. Nowadays, Kazakhstan has one of the highest rates of morbidity and mortality from TB in Europe and the Commonwealth of Independent States [1]. Moreover, Kazakhstan is one of the 24 countries in the European region that has a high number of multiple drug resistant strains [1]. This resistance to one or several forms of treatment occurs when the bacterium develops the ability to withstand antibiotics and relays that ability to its offspring. Drug-resistant TB is difficult and costly to treat, extending the time a person needs to be treated, which sometimes is fatal [2]. Early detection of the disease provides fast recovery and mild course of the disease, reducing the number of fatal cases. The commonly used detection technique based on sputum smear microscopy has low sensitivity [3], whereas other methods based on nucleic acid amplification and microbiological culturing have limitations such as batch-to-batch variations and require expensive devices and reagents [4]. Therefore, it is important to develop a fast, accurate, and inexpensive detection tool specific to TB. TB-specific antigen detection based on abundantly secreted proteins with high levels of immunogenicity could be the most promising approach for such detection tools [5]. Despite existing technologies and advances over the last decades, the development of simple point-of-care test in the near future is still challenging. However, vast advances in nanotechnology and microfluidics led to the development of biosensors [6]. A biosensor is a device that is capable of detecting an analyte, and it consists of a biological recognition system, often called a bioreceptor, and a physicochemical transducer [7]. Depending on the employed physicochemical transducer, TB biosensors can be mass/piezoelectric, electrical, optical, or electrochemical [8,9,10,11,12,13,14]. In this study, specific bioreceptor aptamers that were previously selected against *M. tuberculosis* secreted protein MPT64 were chosen for the detection of actively dividing pathogen [15]. MPT64 is one of the predominant immunogenic secreted *Mtb* antigens and is expressed early when the bacterium is actively dividing [16] and, hence, serves as a biomarker for active TB. The protein was found to be important for mycobacterial survival and persistence in the host cell by deactivating the expression of apoptotic cytokines and promoting the survival and virulence of the mycobacteria [17]. The current TB diagnosis based on MPT64 detection includes skin patch tests, and immunochromatographic, immunohistochemical, and enzyme-linked immunosorbent-based assays (ELISA) [5,18,19,20]. MPT64 detection in the above methods is based on antibody/antigen interaction and, hence, requires the production of temperature-sensitive antibodies. In addition, mycobacteria need to be grown in liquid or solid media; therefore, special laboratory safety precautions and incubation time are required. One potential solution to the limitations of these assays is to use synthetic aptamers instead of antibodies and combine it with more quantitative detection technique such as Electrochemical Impedance Spectroscopy (EIS). Aptamers are single-stranded oligonucleotides (both DNA and RNA) that have abilities to specifically bind to a broad range of targets [21,22,23] with high affinity and specificity [24]. Aptamers have qualities that can overcome some of the disadvantages of antibodies. For example, aptamers are synthesized in vitro from a library that contains large numbers of random sequences. This alleviates use of animals during the selection process compared to antibodies, and their properties can be changed on demand. EIS is a sensitive detection method, where an analyte binding can be recorded within a minute change. The signal can be obtained not only due to a molecular interaction, but also due to an electron and a charge transfer. The use of aptamers as a recognition element and EIS as a detection technique would enable an increase in the accuracy of diagnosis and can be tested directly on the biological sample without growing the bacteria in special media. EIS used in this study integrates an in-house built microfluidic chamber and an interdigitated electrode (IDE). The design and the development of the microfluidic device used in the biosensor development are important. Firstly, careful design should ensure a laminar flow over the sensing surface without disturbing the binding event between the analyte and the ligand. Secondly, results obtained from the sensor must be consistent and reliable. During our preliminary tests, the in-house-made transparent microfluidic cell with embedded reference and counter electrodes showed the presence of small bubbles after some time under continuous filling (Figure 1a). These bubbles deteriorated the overall measurement of the biosensor leading to results with an error message in the screen of the instrument (Figure 1b). These defective results are due to the fact that “wanted” measurement signals have to compete with “unwanted” signals (noise) present in the set-up under testing, such that environmental noise exceeded the useful signals to a certain degree, leading to the error message.

This work aims to assess the filling conditions of the chamber in the micro-device, using Computational Fluid Dynamics (CFD) techniques, to understand the flow conditions that may originate the air bubbles entrainment and to suggest suitable conditions to tackle the problem. Multiphase flow modeling, especially in microfluidics, has evolved dramatically and, despite the difficulty in the treatment of fluid interfaces, current numerical models have succeeded in reproducing with pristine clarity the collapse, breakup, and transport of bubbles and droplets in microchannels and meso-scale flows where laminar flow prevails. Among the existing numerical techniques, the volume of fluid (VOF) method [25], the level set (LS) method [26,27,28,29], the front tracking (FT) method [30,31], and the phase field/Arbitrary Lagrangian–Eulerian (PF-ALE) method are perhaps the most popular ones [32]. The original single fluid, interface tracking, VOF method was developed by Hirt and Nichols and is extensively used in free surface modeling either in its original formulation (e.g., FLOW3D solver) or in the multi-fluid version with a compressive interface algorithm (e.g., ANSYS-CFX and FLUENT). Although, using any model, it is nearly impossible to reach fully accurate curvature and thickness of interfaces, it is possible up to the level of mesh refinement to represent the interfaces while conserving mass by using precise algorithms in the VOF method [25]. The LS method offers the advantage of predicting more accurate curvatures and complex interfaces, but at the expense of mass conservation detriment due to inherent numerical errors, compared with VOF [33]. Nevertheless, significant progresses have been claimed in the mitigation of mass balance errors in LS methods with the introduction of improved algorithms [34,35]; however, none of them have completely succeeded in conserving mass balance at accurate levels as obtained using VOF. In order to overcome the drawbacks of both methods, hybrid formulations such as CLSVOF, MCLS, and ACLSVOF have been developed in which VOF and LS are combined [33]. However, these combinations have introduced rather more complications than advantages, which have made them still unpopular. The FT method is another popular category of multiphase flow modeling in which all phases are handled together by solving a unified set of equations [27]. The PF-ALE method, recently developed by Zheng and Karniadakis, has succeeded in modeling multiphase interface in moving mesh conditions most appropriate in fluid–structure interaction problems [32].

In this investigation, the free surface and air entrainment occurring in the process of filling a micro-biosensor chamber with liquid is modeled using the Eulerian–Eulerian multifluid VOF model with the compressed interface algorithm present in the CFD platform ANSYS-CFX.

## 2. Materials and Methods

### 2.1. Fabrication of the Microfluidic Flow Cell

The microfluidic flow cell was designed based on dimensions of current commercially available microfluidic flow cells. The computer-aided design (CAD) model was generated on ANSYS Design Modeller CAD tool v.2015 and thereafter 3D-printed on Objet-206-Connex printer using Biocompatible Polyjet Photopolymer (StrataSys, Ltd). Reference electrode (Ag-AgCl_2_, Princeton Applied Research) and counter electrodes (Platinum, Princeton Applied Research) were connected to the microfluidic flow cell.

### 2.2. Preliminary Testing of a Biological Sample

The inlet and outlet tubing were connected to the microfluidic chamber and, following common protocols in the operation of similar devices, the infusion with syringe pump (Legato 100, KD Scientific) was only executed after flushing with pure ethanol and 1% SDS (Sigma) for 2 min to remove any contaminant from the tubing and the working area. The buffer was then allowed to flow across the working area at 10 µL/min for at least 20 min to remove the remaining SDS molecules. IDE electrodes (G-IDEAU5, Dropsense) were used as working electrodes. The surface of the IDE was modified by immobilization of aptamers selected against *M. tuberculosis* secreted immunogenic protein MPT64 (EnoGene Biotech Co Ltd, Nanjing, China) [15]. Different concentrations of MPT64 diluted in running buffer were allowed to interact with aptamers on the surface before the signal was measured. Despite following the customary protocol, the presence of air bubbles (Figure 1a) did not allow the correct measurement and motivated an in-deep numerical evaluation of the design.

## 3. CFD Analysis

### 3.1. Governing Equations and Physical Models

An Eulerian–Eulerian multiphase VOF approach with compressive discretization scheme was utilized to model the water filling of the cell chamber, while the air was consequently being removed from it. Mass conservation equations for liquid and air, as well as the volume-fraction weighted Navier–Stokes equations, complete the set of governing equations to be solved under corresponding boundary conditions. Every phase was considered as a continuum through which interpenetration may occur; both phases were assumed as potentially existing in each position of the space, constrained by gravity, friction, and inertial forces. Since the internal surface of the biosensor in contact with the buffer liquid and air was verified to have a contact angle of near 90°, the capillary effect at walls was discarded in the assessment. A final equation was incorporated to enforce that each control volume has a sum of volume fractions of all phases equal to 1.

Governing equations are presented in the next set of equations [36]:

Mass Conservation:

(1) $$\nabla •\left(r_{\alpha }\rho_{\alpha }U_{\alpha }\right)=0.$$

Linear Momentum:

(2) $$\frac{\partial }{\partial t}(\rho U)+\nabla •\left(\left(\rho U⊗U-\mu \left(\nabla U+{\left(\nabla U\right)}^{T}\right)\right)\right){=}_{}\left(B-\nabla p\right).$$

Thus, here,

(3) $$U_{\alpha }=U_{\beta }=U$$

(4) $$p_{\alpha }=p_{\beta }=p$$

(5) $$\rho =\sum_{\alpha =1}^{2}r_{\alpha }\rho_{\alpha }$$

(6) $$\mu =\sum_{\alpha =1}^{Np}r_{\alpha }\mu_{\alpha }$$

and, for the volume fractions,

(7) $$\sum_{\alpha =1}^{2}r_{\alpha }=1$$

where α and β represent the water and air phases, respectively; U is the velocity field; *p* is the pressure field; ρ is the fluid density; μ is the fluid viscosity; and B is the body forces (gravity only in this case). Buoyant force due to density difference of phases and gravity in the z-axis was prescribed. Air and water constant properties at ambient temperature were considered in the analysis; thus, density and viscosity corresponded to the ambient condition.

The Surface Tension Model: In this model, a body force ${\vec{F}}_{S}$ was enforced on the momentum equation at a liquid–gas interface. As an adaptation of the Young–Laplace equation including effects by tangential stresses, the force was calculated by

(8) $${\vec{f}}_{S}=\gamma \kappa +\nabla_{S}\gamma .$$

Then, the force was modified as a continuum per unit volume according to Brackbill et al. [37]:

(9) $${\vec{F}}_{S}={\vec{f}}_{S}\delta_{S}={\vec{f}}_{S}\left|\nabla r_{\alpha }\right|=\gamma \kappa \nabla r_{\alpha }+\nabla_{S}\gamma .$$

In this particular case, ∇_s_γ was zero since Marangoni stresses were not taken into account because the surface tension coefficient, γ, was constant.

The governing equations were discretized using the finite volume method with a second-order upwind scheme in space and second-order Euler-backward time-stepping. The domain was divided into tetrahedral and hexahedral elements and the solution was obtained using the numerical platform ANSYS-CFX v17.2 student version.

The water–air surface tension coefficient was taken as 0.071 N/m, which corresponds to its value at ambient conditions [38]. Initially, the chamber was full of air, and a set of numerical simulations was then performed for water flooding the chamber at volumetric flow rates of 15 µL/min, 100 µL/min, 500 µL/min, and 1000 µL/min. The outlet boundary condition was prescribed as 0 Pa and a zero velocity gradient. The t-step was chosen after a preliminary stability assessment leading to converged and accurate results. Thus, transient simulations with uniform t-steps of 0.01 s were executed to advance towards the quasi-steady solution.

### 3.2. Computational Domain and Boundary Conditions

**Figure 2:**
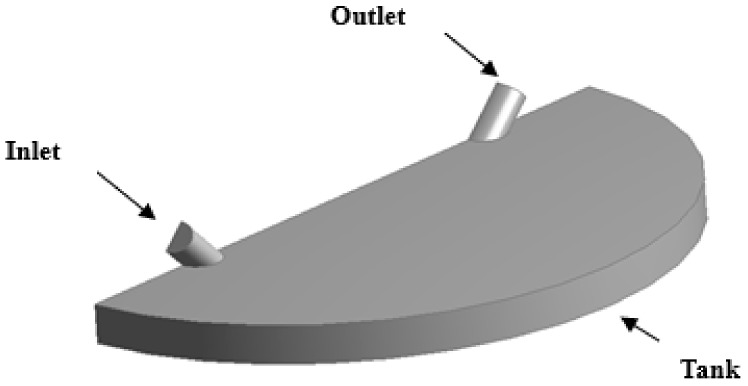
3D view of the computational domain representing half of the microfluidic cell.

Due to memory and CPU-time limitations associated with any simulation, the geometry was halved by cutting through its symmetry plane as shown in Figure 2. The computational domain consisted of inlet and outlet tubing connected to the micro-chamber, in which water and air were expected to interact during the filling process. The dimensions of chamber and connectors were as follows.

*Diameter inlet-outlet connectors:* 0.35 mm

Diameter of tank: 5.2 mm

Length of inlet-outlet connectors: 5 mm

Height of tank: 0.3 mm

### 3.3. Mesh Verification and Boundary Conditions

The computational domain was discretized with tetrahedral, hexahedral, and prismatic elements to adapt better to near-wall regions. Meshes with 54,000 (XX-Coarse), 110,000 (Coarse), 230,000 (Medium), and 494,115 (Fine) nodes were preliminarily evaluated to assess the mesh dependency. In order to perform a mesh sensitivity analysis, one point in a critical location within the micro-tank was selected. The point was located at the middle of the inlet tube and (x,y,z) coordinates: 0.05 mm, −1.06 mm, and 0.10 mm. Then, at a fixed time after water started flowing into the chamber, the air fraction at this point was computed and used as a comparison parameter between contiguous trial meshes. Mesh verification results and deviation between contiguous meshes are shown in Table 1. The medium-size mesh was selected for the remaining of the analysis.

## 4. Results and Discussion

Preliminary experiments on the physical model were performed to replicate and better notice the unwanted air-trapping phenomena. Hence, following cleaning and washing steps at higher flow rates (>1000 µL/min), the protein buffer was allowed to flow across the working area at 10 µL/min. The impedance signal was recorded after injecting MPT64 protein and pausing the flow. The measurement of the signal was possible for some protein concentrations; however, in most cases, it was interrupted due to the visible bubble formations on the IDE electrode surface, as mentioned before and shown in Figure 1. Therefore, for the simulation, the flow rate was chosen as a key input variable since it was expected that the inertia of the incoming liquid would affect the tendency to entrain air as the chamber fills up. A reasonable flow rate range was taken from typically acceptable values used in commercial micro-sensors. Table 2 shows the velocity and theoretical time required to fill the chamber (named “critical time”, hereafter) at each corresponding flow rate.

**Figure 3:**
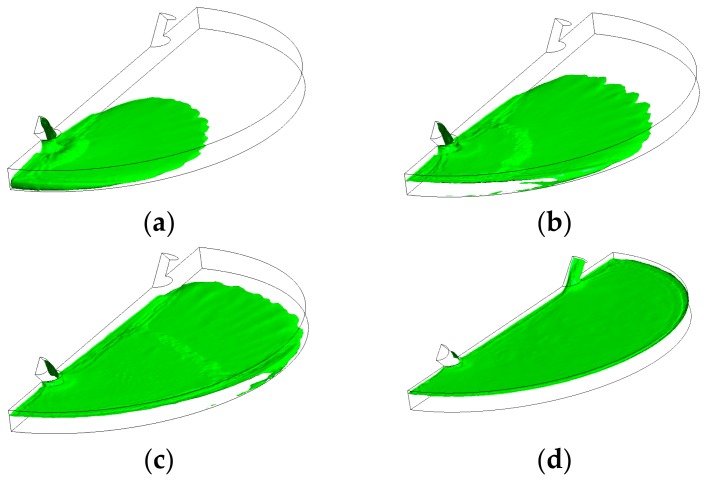
Free-surface during chamber flooding at the following times: (**a**) 5 s < τ_c_; (**b**) 7 s < τ_c_; (**c**) 10 s < τ_c_; (**d**) 15 s > τ_c_, at an inlet liquid flow rate of 15 µL/min (τ_c_ = 12.8 s).

**Figure 4:**
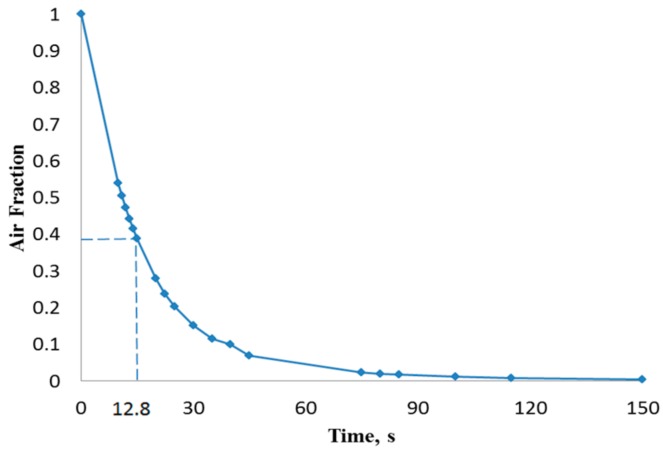
Overall air volume fraction in the chamber vs. filling time at a liquid flow rate of 15 µL/min.

Figure 3 illustrates the filling process in four sequential instants of the first scenario when the volumetric flow rate was 15 µL/min (inlet velocity of 0.0052 m/s). It can be depicted that the flow pattern was quite uniform and no significant free-surface perturbations were observed throughout the flooding process. However, the liquid-free surface depicted moderate waviness, which might drive the phenomenon of air entrainment during the filling process. Figure 4 shows the overall air volume fraction within the micro-device chamber at different times during the filling process at a liquid flow rate of 15 µL/min. Numerical results showed that, at the critical time (12.8 s) at this flow rate, there was still a large overall air volume fraction within the chamber (0.39, indicated in Figure 4 with dashed lines). This result indicates that the filling process was unsuccessful since the flooding of the chamber did not completely remove the air initially present within the chamber.

**Figure 5:**
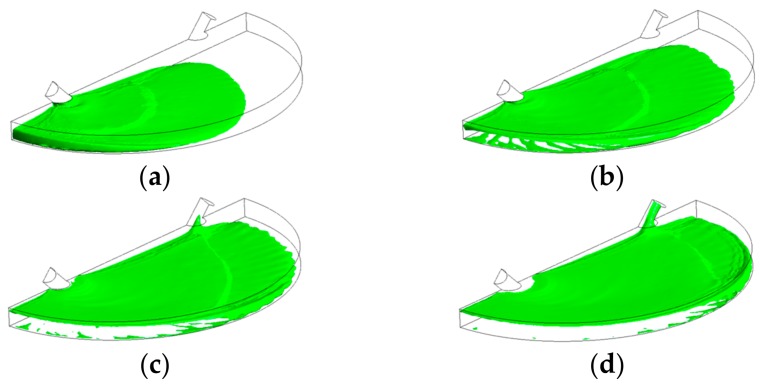
Free-surface during chamber flooding at the following times: (**a**) 0.8 s < τ_c_; (**b**) 1.2 s < τ_c_; (**c**) 1.6 s < τ_c_; (**d**) 2 s ≈ τ_c_, at an inlet liquid flow rate of 100 µL/min (τ_c_ = 1.93 s).

**Figure 6:**
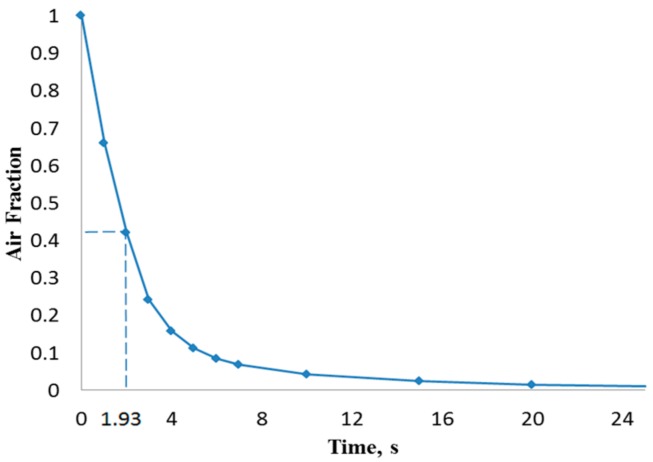
Overall air volume fraction in the chamber vs. filling time at a liquid flow rate of 100 µL/min.

**Figure 7:**
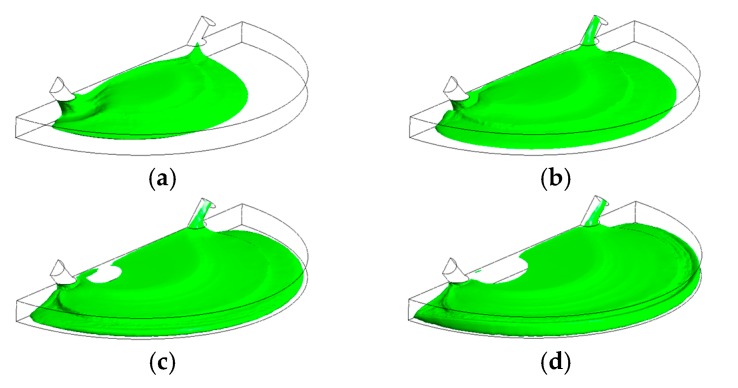
Free-surface during chamber flooding at the following times: (**a**) 0.15 s < τ_c_; (**b**) 0.25 s <τ_c_; (**c**) 0.38 s ≈ τ_c_; (**d**) 0.5 s > τ_c_, at an inlet liquid flow rate of 500 µL/min (τ_c_ = 0.385 s).

**Figure 8:**
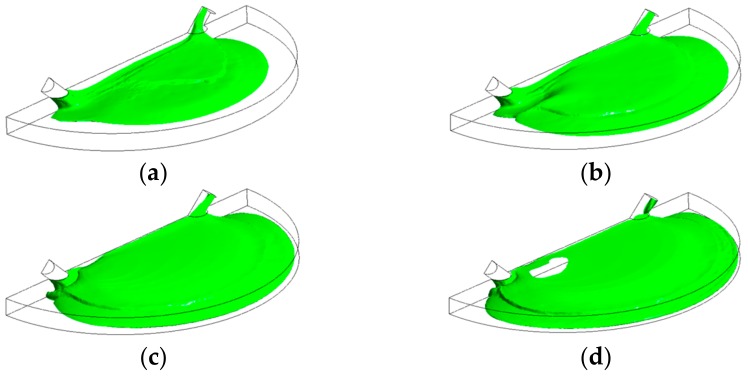
Free-surface during chamber flooding at the following times: (**a**) 0.1 s < τ_c_; (**b**) 0.15 s < τ_c_; (**c**) 0.2 s ≈ τ_c_; (**d**) 0.25 s < τ_c,_ at an inlet liquid flow rate of 1000 µL/min (τ_c_ = 0.193 s).

**Figure 9:**
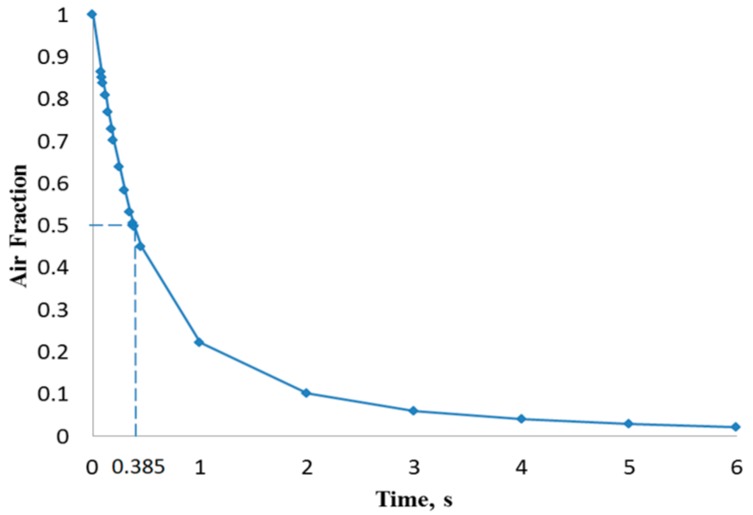
Overall air volume fraction in the chamber vs. filling time at a liquid flow rate of 500 µL/min.

**Figure 10:**
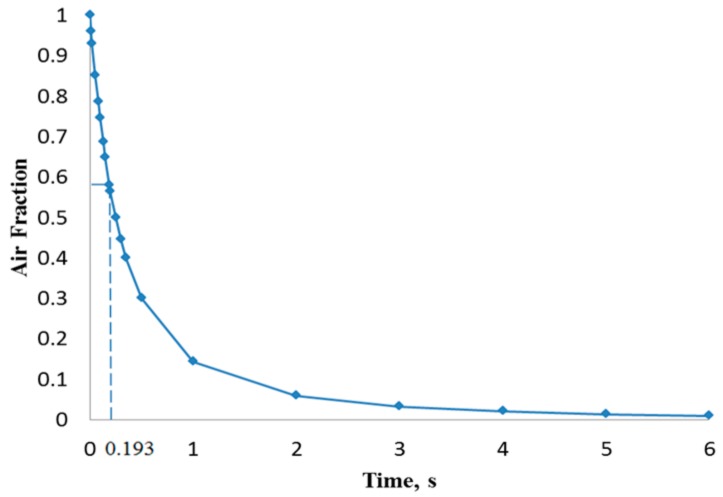
Overall air volume fraction in the chamber vs. filling time at a liquid flow rate of 1000 µL/min.

**Figure 11:**
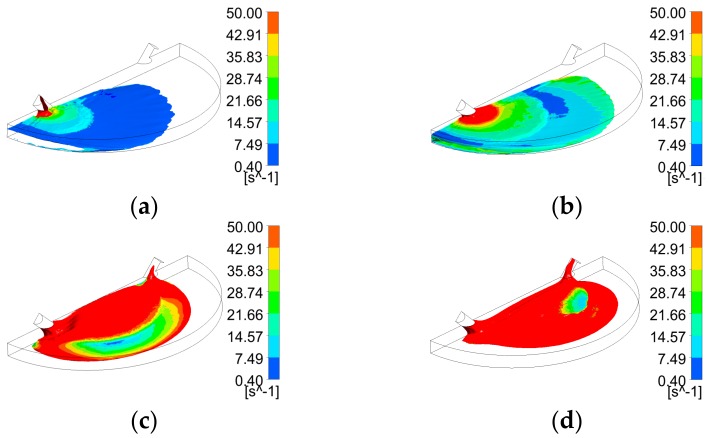
Free surface vorticity of four flooding scenarios during chamber flooding at their half critical times; (**a**) 15 µL/min (6.5 s); (**b**) 100 µL/min (0.96 s); (**c**) 500 µL/min (0.19 s); (**d**) 1000 µL/min (0.1 s).

**Figure 12:**
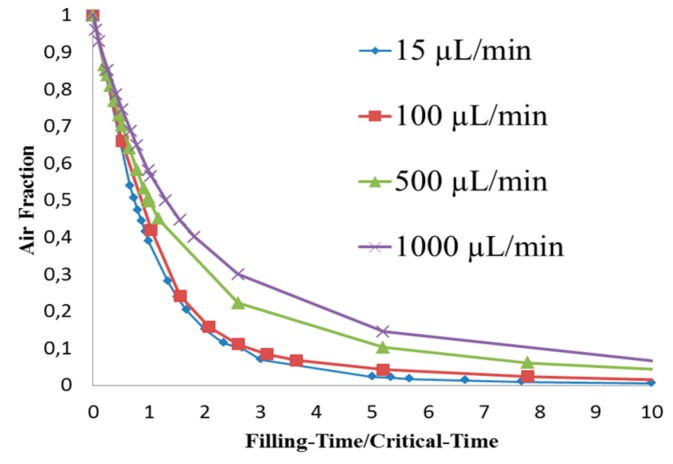
Overall air volume fraction in the chamber vs. Filling-Time/Critical-Time at liquid flow rates of 15, 100, 500, and 1000 µL/min.

Figure 5 shows that the liquid free surface at a flow rate of 100 µL/min causes moderately higher surface instabilities than found for 15 µL/min. This could be explained due to the higher inertia when the liquid entered the chamber with a stronger trend to entrain air initially present in the chamber. According to Figure 6, at critical time at an inlet flow rate of 100 µL/min, a remaining overall air volume fraction of 0.43 was still present in the chamber. A similar pattern can also be observed at flow rates of 500 µL/min (Figure 7) and 1000 µL/min (Figure 8) at which flooding was distinguished to be non-uniform and more agitated. Indeed, for these two flow rates (Figure 9 and Figure 10), overall air volume fractions at critical time were approximately 0.51 and 0.59, demonstrating a growing pattern as the inlet flow rate was increased. This trend can also be observed in Figure 11 where free surface vorticity level intensifies as flow rate increases, which revealed that the level of free surface unsteadiness was a fundamental factor in the presence of air entrainment. Figure 12 illustrates the overall air volume fraction within the chamber versus the ratio of critical time and filling-up time. It can be clearly seen that at a ratio of 1, the inlet flow rate of 15 µL/min led to the lowest air volume fraction in the chamber, which indicates that, among the explored flow rates, this one provided the condition most effective for flooding the chamber; therefore, for practical conditions, it should be recommended.

**Figure 13:**
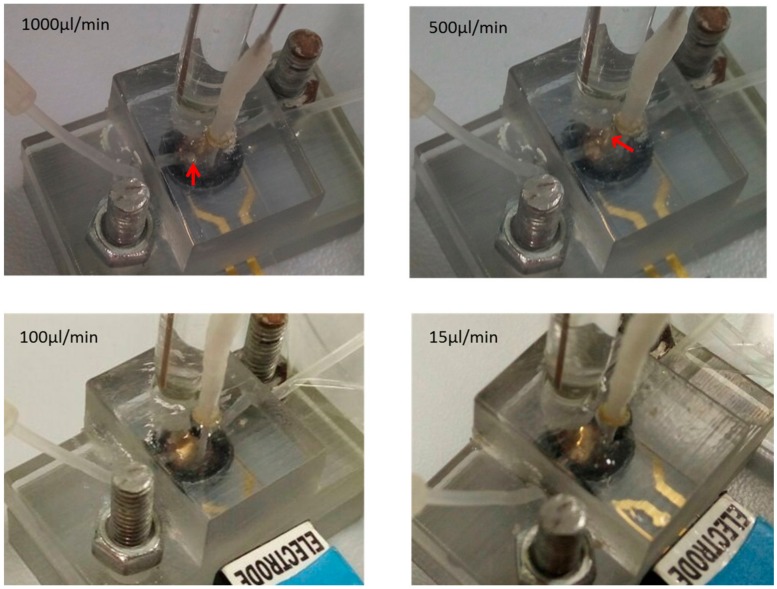
Assembled microfluidic devise with reference, counter, and working electrodes. The red arrow shows the area where air bubbles formed during the course of the experiment at respective flow rates 1000, 500, 100, and 15 µL/min.

Preliminary experimental validation of the model was carried at four different flow rates after injecting the buffer into the chamber until the chamber was fully filled. Then, injection was set at 10 µL/min and the buffer allowed to flow across the working area for at least 10 min. This step was performed to allow the protein to bind to specific aptamers and remove the unbound proteins from the surface. Then, the injection was paused for 4 min (the measurement time required for EIS), and the formation of air bubbles was monitored and captured using the camera. Figure 13 shows the captured images at respective flow rates. The entrapment of air bubbles was clearly seen after the injection at 1000 µL/min and 500 µL/min (Figure 13, see the direction of indicated red arrows). The large air bubble was formed at the base of the injection tube at 1000 µL/min, whereas smaller air bubbles were found in the middle of the working area at 500 µL/min. There was no air bubble seen at 100 µL/min and 15 µL/min flow rates. These results indicate the importance of the flow rate during the initial chamber filling process. Higher flow rates (1000 µL/min, 500 µL/min) induced the air entrapment within the chamber. This also shows the importance of the flow rate during the chamber cleaning steps, which might have an effect on the following measurement steps.

**Figure 14:**
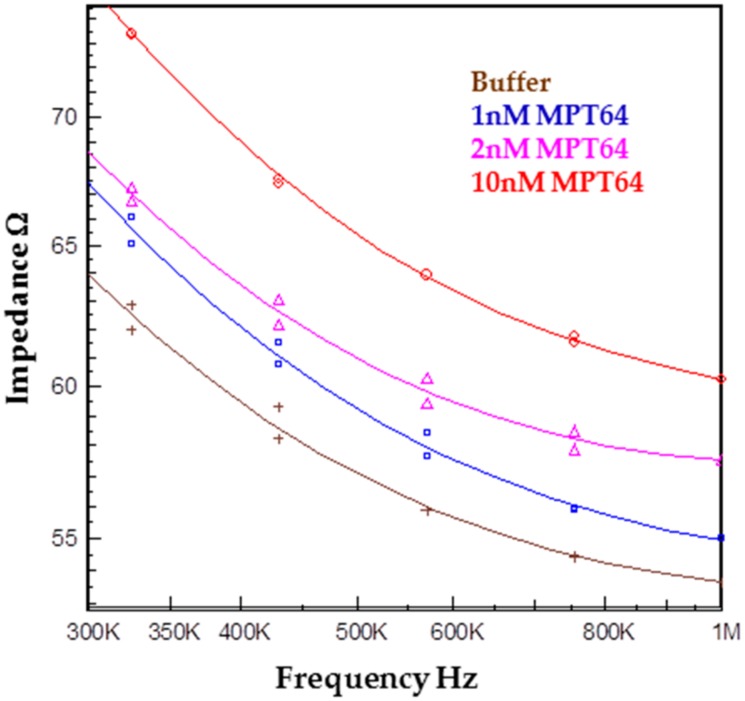
Impedance magnitude data for the detection of the target MPT64 at different concentrations of 1, 2, and 10 nM. The impedance magnitude increased with an increase in the target concentration.

In order to validate that the device can be used as a part of the biosensor, the functionalized IDE electrode with aptamers was inserted into the chamber and connected to the instrument. The chamber was first filled at 100 µL/min with the running buffer. Then, the flow rate was decreased to 15 µL/min, and different concentrations (1, 2, and 10 nM) of MPT64 were allowed to interact with the functionalized surface. The signal was recorded after the incubation of each MPT64 concentration. Air bubbles did not interrupt signal recording and, hence, no error message was received from the instrument. Figure 14 shows the impedance magnitude at different MPT64 concentrations. The binding between aptamer and the target caused an increase in impedance as compared to the running buffer at higher frequencies, i.e., above 300 KHz. The impedance increase was likely caused by the blockage of the flow of ions between the electrode fingers due to the binding of the target onto to the aptamer-modified surface.

## 5. Conclusions

CFD simulations of the flooding process occurring in the chamber of a micro-biosensor under different filling flow rates were presented. Second-order space discretization scheme and second-order Euler-backward time stepping on a multiphase VOF finite volume method were used to solve the set of governing equations, including surface tension effects. Capillary effects related to surface wettability were neglected due to the near-to 90° existing contact angle. Four different water inlet flow rates were explored searching for free-surface waviness and other phenomena responsible of promotion of air entrainment observed during the experimental process. Numerical results showed that, as inlet flow rate increased, more air was trapped within the biosensor chamber. At critical time 12.8 s (theoretical time needed to complete filling) for the scenario with 15 µL/min, the overall air volume fraction remaining in the chamber was 39%. For the other three scenarios (100, 500, and 1000 µL/min) at their critical times (1.926, 0.385, and 0.193 s, respectively), the overall air volume fractions were 43%, 51%, and 59%, respectively. Therefore, it is recommended that the micro-biosensor chamber be filled at the lowest flow rate typically used in these applications in order to diminish the possibility of air entrainment and consequential damage of the measurement capability of the micro-device.

## Figures and Tables

**Table 1:** Mesh dependency analysis.

	Mesh (Feature)	Air Fraction	Deviation, %
1	54,000 (XX-course)	0.240	
2	110,000 (Coarse)	0.299	24.35
3	230,000 (Medium)	0.329	9.94
4	494,115 (Fine)	0.332	0.91

**Table 2:** Flow rate, inlet velocity, and critical time for explored scenarios.

Flow Rate, (µL/min)	Inlet Velocity (m/s)	Critical Time, τ_c_ (s)
15	0.0052	12.8
100	0.0347	1.93
500	0.173	0.385
1000	0.346	0.193
